# Pro-Fibrotic Phenotype in a Patient with Segmental Stiff Skin Syndrome via TGF-β Signaling Overactivation

**DOI:** 10.3390/ijms21145141

**Published:** 2020-07-20

**Authors:** Carmela Fusco, Grazia Nardella, Bartolomeo Augello, Francesca Boccafoschi, Orazio Palumbo, Luca Fusaro, Angelantonio Notarangelo, Raffaela Barbano, Paola Parrella, Giuseppina Annicchiarico, Carmela De Meco, Lucia Micale, Paolo Graziano, Marco Castori

**Affiliations:** 1 Division of Medical Genetics, Fondazione IRCCS-Casa Sollievo della Sofferenza, San Giovanni Rotondo, 71013 Foggia, Italy; g.nardella@operapadrepio.it (G.N.); b.augello@operapadrepio.it (B.A.); o.palumbo@operapadrepio.it (O.P.); a.notarangelo@operapadrepio.it (A.N.); l.micale@operapadrepio.it (L.M.); m.castori@operapadrepio.it (M.C.); 2 Health Science Department University of Piemonte Orientale, Novara 28100, Italy; francesca.boccafoschi@med.uniupo.it; 3 Tissuegraft srl, 28100 Novara, Italy; luca.fusaro@med.uniupo.it; 4 Laboratory of Oncology, Fondazione IRCCS-Casa Sollievo della Sofferenza, San Giovanni Rotondo, 71013 Foggia, Italy; r.barbano@operapadrepio.it (R.B.); pparrella@operapadrepio.it (P.P.); 5 Coordinamento Regionale Malattie Rare Puglia, Agenzia Regionale per la Salute ed il Sociale, Bari, 70132 Puglia, Italy; annicchiarico.giuseppeina@gmail.com; 6 Division of Pediatrics, Fondazione IRCCS-Casa Sollievo della Sofferenza, San Giovanni Rotondo, 71013 Foggia, Italy; c.demeco@operapadrepio.it; 7 Unit of Pathology, Fondazione IRCCS-Casa Sollievo della Sofferenza, San Giovanni Rotondo, 71013 Foggia, Italy; p.graziano@operapadrepio.it

**Keywords:** fibrosis, losartan, pathogenesis, stiff skin syndrome, TGF-β

## Abstract

Transforming growth factor β (TGF-β) superfamily signaling pathways are ubiquitous and essential for several cellular and physiological processes. The overexpression of TGF-β results in excessive fibrosis in multiple human disorders. Among them, stiff skin syndrome (SSS) is an ultrarare and untreatable condition characterized by the progressive thickening and hardening of the dermis, and acquired joint limitations. SSS is distinct in a widespread form, caused by recurrent germline variants of *FBN1* encoding a key molecule of the TGF-β signaling, and a segmental form with unknown molecular basis. Here, we report a 12-year-old female with segmental SSS, affecting the right upper limb with acquired thickening of the dermis evident at the magnetic resonance imaging, and progressive limitation of the elbow and shoulder. To better explore the molecular and cellular mechanisms that drive segmental SSS, several functional studies on patient’s fibroblasts were employed. We hypothesized an impairment of TGF-β signaling and, consequently, a dysregulation of the associated downstream signaling. Lesional fibroblast studies showed a higher phosphorylation level of extracellular signal-regulated kinase 1/2 (ERK1/2), increased levels of nuclear factor-kB (NFkB), and a nuclear accumulation of phosphorylated Smad2 via Western blot and microscopy analyses. Quantitative PCR expression analysis of genes encoding key extracellular matrix proteins revealed increased levels of *COL1A1*, *COL3A1*, *AGT*, *LTBP* and *ITGB1*, while zymography assay reported a reduced metalloproteinase 2 enzymatic activity. In vitro exposure of patient’s fibroblasts to losartan led to the partial restoration of normal transforming growth factor β (TGF-β) marker protein levels. Taken together, these data demonstrate that in our patient, segmental SSS is characterized by the overactivation of multiple TGF-β signaling pathways, which likely results in altered extracellular matrix composition and fibroblast homeostasis. Our results for the first time reported that aberrant TGF-β signaling may drive the pathogenesis of segmental SSS and might open the way to novel therapeutic approaches.

## 1. Introduction

The hyperactivation of transforming growth factor beta (TGF-β) mediated signaling pathways stimulates fibrosis and, as such, it has been demonstrated or postulated in multiple acquired and congenital human disorders. Among them, stiff skin syndrome (SSS, MIM 184900) is an ultrarare and untreatable disorder characterized by generalized or localized progressive non-inflammatory fibrosis of the skin, which turns harder and thicker. Joint limitations, lipodystrophy, and muscle weakness are common satellite findings. SSS was first described in 1971 [1], and about 60 cases have been reported to date [1,2,3,4,5,6,7,8,9,10,11]. While the generalized form of SSS is a widespread and bilateral disorder, localized or segmental SSS has unilateral or monomelic presentation with consequences limited to the affected body segment. Loeys et al. demonstrated germline heterozygous variants in the Fibrillin 1 gene (*FBN1*) as responsible for generalized SSS [12]. Although reported in 4 families only, genotype–phenotype correlations seem strict, as SSS-specific *FBN1* variants alter both microfibrillar assembly and microfibrill–integrin interactions, which contribute to TGF-β signaling dysregulation. Accordingly, the authors demonstrated a dramatic increased deposition of collagen, elastin, and fibrillin 1 in patients’ skin [12].

Conversely, the molecular basis of segmental SSS remains elusive, and this implies that segmental SSS is often misdiagnosed or underdiagnosed. Some researchers suggested that segmental SSS might be caused by somatic variants in *FBN1*, but this hypothesis was never investigated. Fragmented insights in the pathogenesis of SSS come from early works, which demonstrate an increased collagen synthesis in the dermis [10], as well as an excess of selected circulating cytokines including TNFα, interleukin (IL)-6, and TGF-β2 [11,13]. As a consequence, SSS is without a cure. Single case reports suggest improvement with an oral intake of mycophenolate mofetil [14], secukinumab [15], and losartan [16], the latter already known as a treatment resource for other disorders related to transforming growth factor-β (TGF-β) signaling dysregulation, such as Marfan syndrome [17]. All these reports are preliminary observations without any experimental proof of the presumed biological rationale.

TGF-β is a key profibrotic multifunctional cytokine involved in many human diseases. The TGF-β signal exerts its biological effects via the TGF-β/Smad-mediated and non-Smad–mediated signaling pathways [18]. Elevated levels of TGF-β, either locally or systemically, can result in the activation of their associated downstream signaling. Acting through the Smad2 axis, TGF-β isoforms drive the expression of key extracellular matrix (ECM) profibrotic genes, including collagens [19], fibronectin [20], and latent transforming growth factor beta binding protein (*LTBP*) [21]. On the other hand, the TGF-β–induced non-Smad signaling leads to the activation of extracellular signal-regulated kinase 1/2 (ERK1/2), Ras Homolog Family Member A (RhoA), and PI3K/AKT/mTOR (PI3/Akt) pathways, which induce tight junction disassembly, cytoskeletal rearrangement, increased cell motility, and β-catenin nuclear translocation [22,23,24].

In this work, we demonstrated overactivation of the TGF-β signaling pathways in the affected skin of a 12-year-old individual with segmental SSS. To better delineate the biological mechanism(s) that leads to disease, we explored molecular and cellular aspects on primary fibroblast patient lines by employing multiple investigative strategies. In detail, we explored the expression of pro-fibrotic genes, the matrix metalloproteinase-2 (MMP2) enzymatic activity, cell motility, and proliferation, as well as the in vitro effects of losartan exposure on the TGF-β levels in patient’s fibroblasts.

## 2. Results

### 2.1. Clinical and Histological Findings

**Figure 1:**
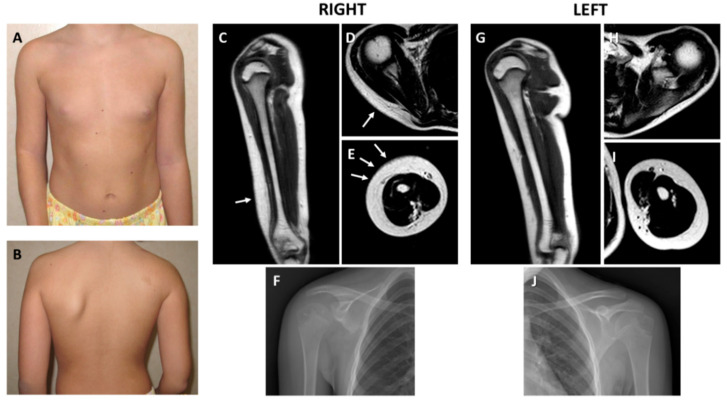
Clinical and radiologic features. (**A**) Front view showing limitation of the extension of the right elbow with accentuation of the cutaneous articular fold. (**B**) Back view demonstrating hypoplasia of the right scapula; note a small café-au-lait spot in the upper scapular region. Radiological findings of the right upper limb. (**C**) Thickening of the dermis of the lateral side of the arm at Magnetic Resonance Imaging (MRI) coronal view (arrow). (**D**) Thickened dermis at the scapular level (arrow). (**E**) Thickened dermis surrounding the arm muscles at axial view (arrows). (**F**) Plain radiograph documenting hypoplasia of the inferior part of the right scapula. Radiological findings of the left upper limb. Comparison with similar MRI sections of the affected (right) side demonstrated normal representation of the dermis at coronal (**G**), and upper (**H**) and lower (**I**) axial views. Muscles are underdeveloped in the right arm. (**J**) Normal left scapula at standard X-rays.

**Figure 2:**
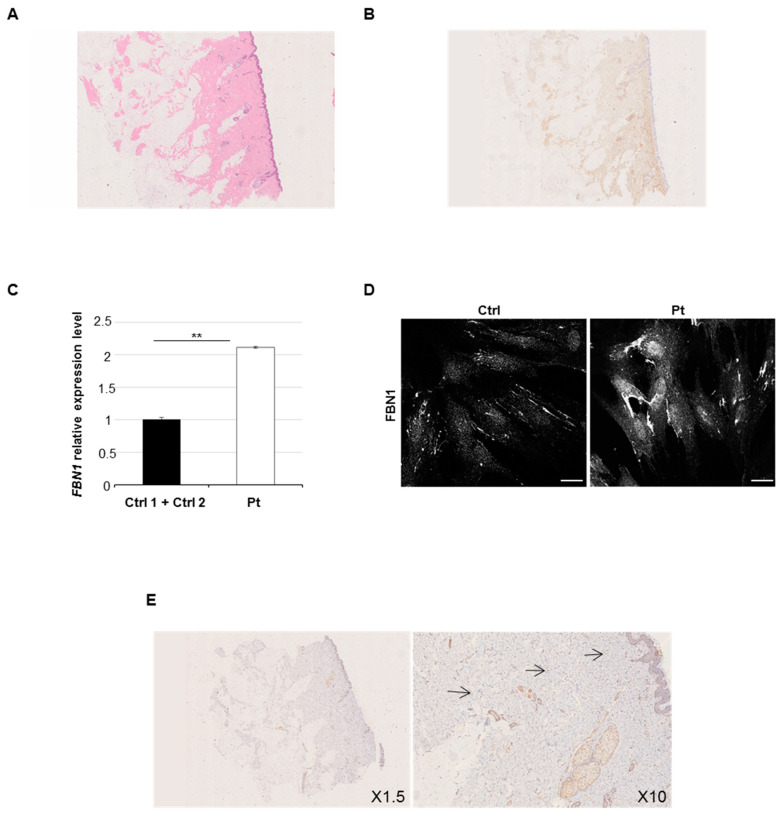
Immunohistochemistry and Fibrillin 1 gene (*FBN1*) analysis. Dermal histology: (**A**) diffuse sclerosis of the dermis and subcutaneous fibroadipose tissue (hematoxylin–eosin staining, H&E X1.5); (**B**) collagen fibers deposition into subcutanous adipose tissue highlighted by trichrome staining and immunoreactivity for anti-CD34 Ab (X1.5). (**C**) Quantitative Real Time PCR (qPCR) was performed to measure *FBN1* expression in patient and controls. Scale bars represent standard errors. **p < 0.03, Student’s *t*-test. Ctrl, control; Pt, patient. (**D**) Confocal study showed an excessive microfibrillar aggregates in patient’s fibroblasts compared to controls by staining with anti-fibrillin 1 antibody (Ab). The panel reports only one representative image from one of the two control lines. Scale bar = 1 μm (white line). (**E**) Immunostaining with anti-fibrillin Ab (X1.5 and X10 objectives) displayed collagen fibers expression in patient’s skin biopsy. Arrows indicate the staining with anti-fibrillin Ab.

The patient is a 12-year-old female, unique child of a 42-year-old father and a 39-year-old mother. Parents were healthy and unrelated. She was born at term by uneventful pregnancy and vaginal delivery. Neonatal presentation, psychomotor development, and mental function were normal. At 6 years of age, the parents first noted the thickening and hardening of the skin over the right scapula and arm. In the ensuing years, the lesion worsened with the progressive involvement of the surrounding skin, as well as with acquired limitations of the underlying joints. Serum markers of inflammation, autoantibodies, and immunoglobulins were repeatedly investigated but always had normal values. Plain radiographs excluded articular malformations and degenerative disease, as well as any sign of endosteal or periosteal hyperostosis. There was hypoplasia of the right scapula due to the underdevelopment of its inferior part, which was not compatible with Sprengel deformity, and generalized osteopenia was evident at standard X-rays. Any underlying disorders of the bone turnover and metabolism were excluded. At 9 years of age, soft tissue magnetic resonance imaging confirmed thickening of the dermis at the lateral side of the right arm and over the shoulder. A skin biopsy carried out in another center concluded for a connective tissue nevus. Nevertheless, the lesion had a progressive course with worsening of the local manifestations. At physical examination, at 11 years of age, a plaque of indurated and finely hypertrichotic skin was appreciable at the right shoulder and external surface of the arm. Severe limitation of the external rotation and elevation of the shoulder, as well as of the forearm extension, was evident. Pronosupination of the right forearm was impossible. The mobility of all other joints was within the normal range. Hypoplasia of the right scapula was clinically evident. A comparison of the clinical and radiological findings is reported in Figure 1A–J. A second skin biopsy showed an increased deposition of collagen fibers without surrounding infiltration of the papillary dermis at hematoxylin and eosin staining (Figure 2A) and an increase of the connective tissue density at trichrome staining highlighted also by CD34 immunoreactivity (Figure 2B). The overlaying epidermis and dermal vessels were normal. The non-congenital and progressive nature of the lesion, absence of serum and local signs of inflammation and immune activation, and histologic evidence of thickening of the dermis allowed the diagnosis of localized or segmental SSS.

### 2.2. Germline and Somatic FBN1 Variants Analysis

Since variants affecting the integrin-binding TB4 domain of Fibrillin 1 were associated to generalized SSS [12], we analyzed the patient’s DNA from peripheral blood by employing a next-generation sequencing (NGS) targeted-gene panel also including *FBN1* as well as a wider array of TGF-β pathway-associated genes.

The NGS analysis of possible variants in causative genes was carried out considering allelic frequency (1000 Genomes, dbSNP 151, GO-ESP 6500, ExAC, TOPMED, GnomAD, NCI60, COSMIC), the pathogenicity score came from different predictors programs (SIFT, Polyphen2, LRT, MutationTaster, MutationAssessor, FATHMM, PROVEAN, VEST3, MetaSVM, MetaLR, M-CAP, CADD, DANN, fathmm-MKL, Eigen, GenoCanyon) or from specific databases (ClinVar, HGMD, LOVD). We analyzed the coding region and exon–intron junctions in FBN1 and all other genes (the full list of analyzed genes are reported in “Methods”). However, the NGS analysis of DNA’s patient excluded any candidate variants in all reported genes.

In order to exclude any somatic mosaic variants in *FBN1*, we analyzed DNA extracted from patient’s fibroblasts followed by NGS analysis by applying a sensitive sequencing assay with mean coverage depth per base. In addition, in this case, the analysis results were negative. Additionally, analysis of intragenic deletions or duplications via CytoScan XON excluded any copy number variations (CNVs) in *FBN1* and all TGF-β signaling-associated genes, in particular all signaling-related disease-causing genes included in the NGS panel (see “Methods”).

Multiple lines of evidence have suggested a contribution of the connective tissue protein Fibrillin 1 to the pathogenesis of profibrotic phenotypes. Therefore, although we did not find any causative variant in *FBN1*, we explored the *FBN1* transcriptional level in the patient’s skin. qPCR analysis revealed that the patient’s fibroblasts expressed a higher level of *FBN1* transcripts compared to controls (Figure 2C). Immunofluorescence (Figure 2D) and immunohistochemistry (Figure 2E) assays with anti-Fibrillin 1 Ab on patient’s fibroblasts and dermal biopsy, respectively, confirmed an intracellular accumulation of detected protein.

### 2.3. Altered Smad- and Non-Smad-Dependent TGF-β Signaling Pathways

**Figure 3:**
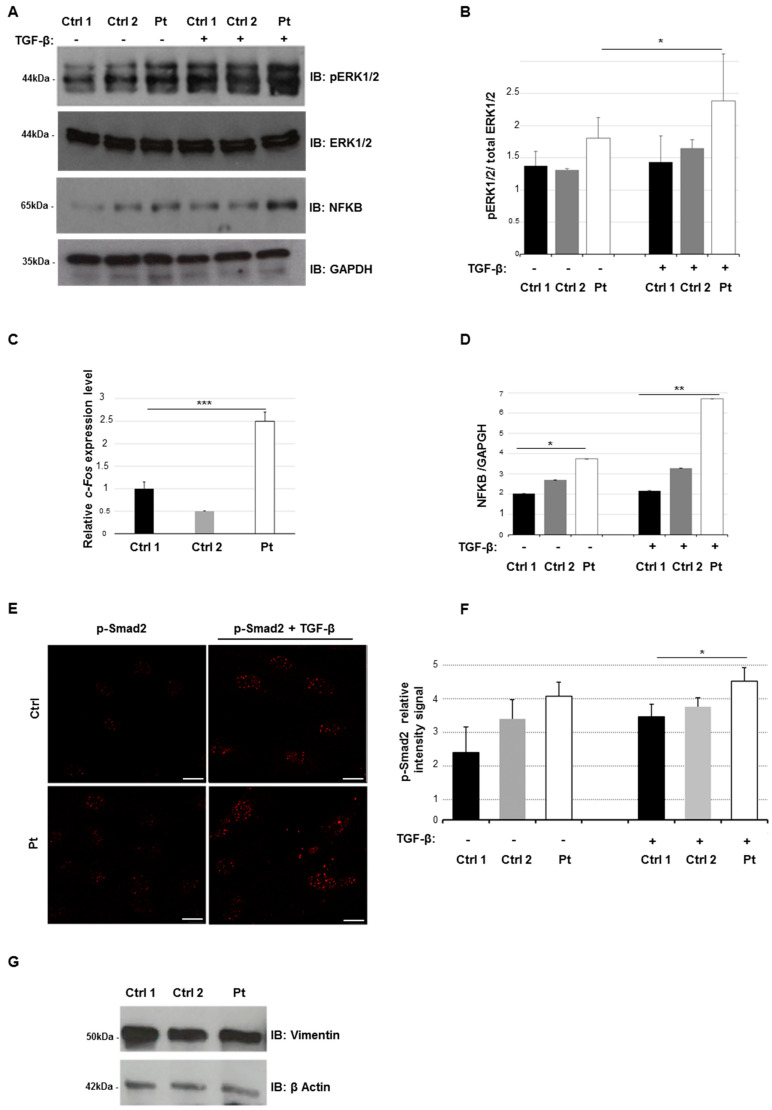
Transforming growth factor β (TGF-β) non-canonical and canonical signaling anomalies. (**A**) Cell lysates were obtained from skin fibroblasts from patient (Pt) and controls (Ctrl) after stimulation with TGF-β (10 ng/mL) during 2 h in serum-free medium. Whole protein lysates were separated on 10% SDS-gel and subjected to immunoblotting with anti-ERK1/2 (extracellular signal-regulated kinase 1/2), anti p-ERK1/2, anti-NFKB (nuclear factor-kB) and anti-GAPDH Abs. (**B**) Levels of phospho–ERK1/2 were quantified by densitometry using IMAGEJ analysis software. Relative p-ERK1/2 levels were normalized compared to total ERK1/2 levels. GAPDH was used as internal control. Graphs show averages calculated on three different experiments, and scale bars represent standard errors. Values are expressed as mean ± SEM (* *p* < 0.05, *n* = 3). (**C**) qPCR was performed to measure the *c-Fos* endogenous expression in patient in controls 1 and 2. The expression levels of control 2 and patient were normalized to control 1. Three independent experiments in triplicate for each replicate were carried out. Scale bars represent standard errors. *** *p* < 0.01, Student’s *t*-test. (**D**) Levels of NFKB were quantified by densitometry using IMAGEJ analysis software. Graphs show averages calculated on three different experiments and scale bars represent standard errors. Values are expressed as mean ± SEM (* *p* < 0.05, ** *p* < 0.03, *n* = 3). (**E**) Confocal study showed the nuclear localization of p-SMAD2 in fibroblasts from patients and controls, with or without TGF-β stimulation (10 ng/mL) during 2 h in serum-free medium and staining with anti-p-Smad2 antibody. The panel reported only one image from a control line as an example of representative control lines. Scale bar = 1 μm (white line). (**F**) After acquisition, for all images, we analyzed the intensity of Alexa Fluor 568 signal, measuring the relative intensity of pixels representative for each region of interest (ROI) corresponding to a single cell by LAS-X software. The graph reports means ± s.d. of p-Smad2 intensity values from 100 cells for each experiment (* *p* < 0.05, *n* = 3). (**G**) Cell lysates were obtained from skin fibroblasts of patients and two controls. Whole protein lysates were separated on 10% SDS-gel and subjected to immunoblotting with anti-Vimentin and anti-β Actin Abs.

In order to explore the alteration of TGF-β signaling in our patient, we evaluated the activation level of non-Smad- and Smad-dependent signaling. Thus, we measured the phosphorylation level of ERK1/2, activated downstream of the TGF-β receptor complex [25] with and without TGF-β stimulation. The phosphorylation level of ERK1/2 (p-ERK1/2) in patient’s fibroblasts after TGF-β activation was higher than control cells as well (Figure 3A,B). This molecular evidence was supported by profiling the endogenous expression level of *c-Fos*, which is a well-known direct transcriptional target of the ERK1/2 axis [26]. We found that *c-Fos* expression was significantly overexpressed in the patient than two controls (>1.5 fold) (Figure 3C).

As ERK1/2 activation controls the levels of the transcription factor nuclear factor-kB (NFkB) [27], we next explore the effect of ERK1/2 activation on NFkB levels by performing an immunoblotting analysis. We found significant increased levels of NFkB in patient’s fibroblasts compared to controls, even upon TGF-β stimulation (Figure 3A,D).

To better confirm an impairment of TGF-β signaling in our patient, we evaluated the nuclear level of p-Smad2 protein in patient’s fibroblasts by confocal analysis. We observed a wider nuclear concentration of p-Smad2 in patient’s cells than controls, with or without TGF-β stimulation (Figure 3E,F). The increased nuclear intensity signal relative of p-Smad2 staining suggests a hyperactivation of TGF-β Smad2 signaling in our patient.

These data suggest that in fibroblasts from localized SSS, there is a wide activation of Smad and non-Smad signaling pathways, downstream of TGF-β. Since some published data show an abundance of myofibroblasts in skin biopsies from SSS patients [28], we assessed the level of myofibroblasts-like cells in our fibroblast cultures by using the myofibroblast marker vimentin. Our Western blot analysis did not reveal a significant difference in the vimentin protein expression levels in patient’s cells compared to controls. These data suggest that our cell cultures contain a comparable fraction of myofibroblasts-like cells (Figure 3G).

### 2.4. Increased Expression Profile of Key Pro-Fibrotic Molecules

**Figure 4:**
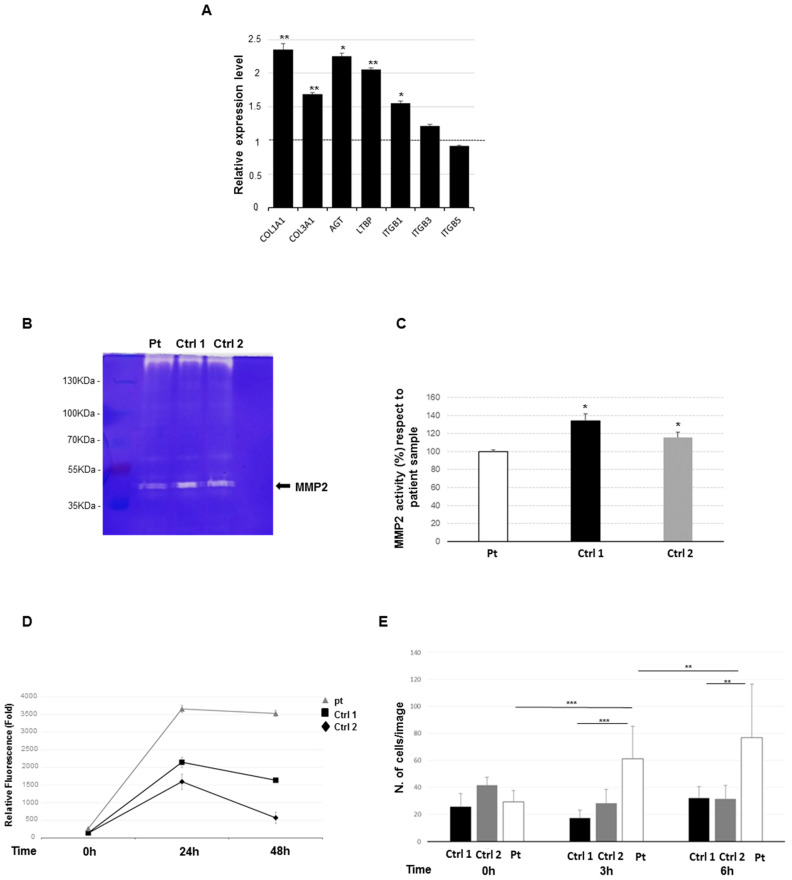
**Analysis of fibrotic profile and proliferation study.** (**A**) Trans-activation of pro-fibrotic genes was assessed by qPCR monitoring endogenous levels of *COL1A1*, *COL3A1*, *AGT*, *LTBP1*, *IGTB1*, *IGTB3,* and *IGTB5*. The fold change value is relative to the mean expression levels of two controls, which were set as value 1 and showed by a dashed line on the histogram. Scale bars represent standard errors. Values are expressed as mean ± SEM (* *p* < 0.05, ** *p* < 0.03, *n* = 3). (**B**) Representative gelatin zymography for matrix metalloproteinase-2 (MMP2), from patient (Pt) and two control (Ctrl) lines. The molecular weight is indicated. (**C**) Quantification of MMP2 activity based on zymography assay. Experiments were performed in triplicate, * = *p* < 0.05. (**D**) Graphic result of viability assay (PrestoBlu) in patient and controls cells. The rate of proliferation viability was annotated for 48 h. Results were statistically analyzed by ANOVA test. (**E**) Quantitative analysis of cell migration is given as the mean ± SD of cells per field. Results were normalized against the control gel condition. ** *p* < 0.03, *** *p* < 0.01.

Acting through the Smad2 axis, TGF-β drives the expression of ECM profibrotic genes, such as collagens [19] and fibronectin [20]. Consequently, we hypothesized a de-regulation of the expression of profibrotic genes in patient’s cells. Hence, we analyzed the expression profile of selected genes that encode for key factors of wound fibrosis [29,30], including *COL1A1*, *COL3A1*, *AGT*, *LTBP*, *ITGB1*, *ITGB3,* and *ITGB5*. Using qPCR, we observed a significant overexpression of *COL1A1*, *COL3A1*, *AGT*, *LTBP*, *ITGB1*, and *ITG3,* while the expression levels of *ITGB5* results were grossly unchanged (Figure 4A). This finding supports the hypothesis that the overexpression of key profibrotic ECM molecules might play a role in patient’s dermal fibrosis.

### 2.5. Reduced Metalloproteinase 2 Activity

We speculated that the dermal fibrosis observed in the patient might be also due, in part, to an impaired degradation of ECM proteins. To explore this suggestion, we evaluated the enzymatic levels of matrix metalloproteinase-2 (MMP2), which is the major extracellular matrix remodeling enzyme, involved in different fibrotic disorders [31,32], by a gelatin zymography. The total MMP2 enzymatic activity in patient’s fibroblasts showed a significant reduction than controls (Figure 4B,C). In detail, the histogram reported a significant basic difference in MMP2 enzyme activity levels in the patient compared to control cell lines. These data suggest that the reduction of enzymatic activity of MMP2 might contribute to the accumulation of ECM proteins in our patient, as previously reported in other fibrotic diseases [31,33].

### 2.6. Increased Cell Proliferation and Migration

To test the hypothesis that the documented TGF-β overactivation can affect the cell proliferation and migratory capacity in our patient, cell viability and collective cell migration assays were carried out on patients’ lesional fibroblasts. The cell viability assay showed an increased cell proliferation rate of patient’s cell line compared to controls. The rate of proliferation has been annotated up to 2 days (Figure 4D). The longitudinal evolution of cell proliferation was significantly different (ANOVA *p* < 0.0001; pairwise comparisons: Pt versus Ctrl 1 *p* < 0.0001; Pt versus Ctrl 2 *p* = 0.0020). Nevertheless, at 48 h, we observed a lower decrease in cell growth rates probably due to a cell confluence. The Transwell collective cell migration assay demonstrated an increase of the migrating cell number in patient’s fibroblasts compared to controls at 3 and at 6 h (Figure 4E). The difference of the average number of cells on the underside of the membrane was statistically significant by comparing the patient’s and controls’ cell lines during the time of analysis. These findings suggest that in patient’s cells, altered TGF-β signaling pathways could promote cell proliferation and migration.

### 2.7. Losartan Reduces the Activation Status of TGF-β Signaling Pathways in SSS Cells

**Figure 5:**
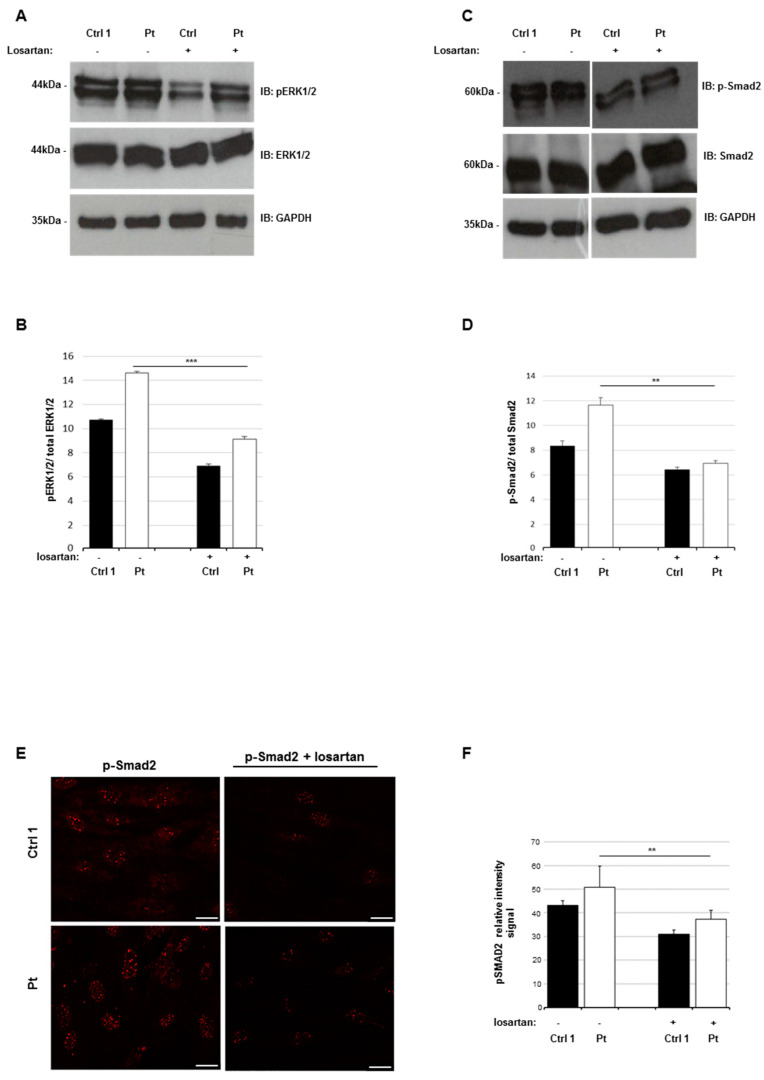
Losartan reduces the TGF-B signaling in patient’s lesional fibroblasts. (**A**) Cell lysates were obtained from skin fibroblasts after losartan incubation (200 µM) for 14 days. Whole protein lysates were separated on 10% SDS-gel and subjected to immunoblotting with anti-ERK1/2, anti p-ERK1/2, and anti-GAPDH Abs. GAPDH was used as internal control. (**B**) Levels of p-ERK1/2 and ERK1/2 were quantified by densitometry using IMAGEJ analysis software. Relative p-ERK1/2 level was normalized compared to ERK1/2 level. Values are expressed as mean ± SEM (*** *p* < 0.01, n = 3). (**C**) Cell lysates were obtained from skin fibroblasts after losartan incubation (200 µM) for 14 days. Whole protein lysates were separated on 10% SDS-gel and subjected to immunoblotting with anti-p-Smad2, anti p-Smad2 and anti-GAPDH Abs. GAPDH was used as internal control. (**D**) Levels of p-Smad2 and Smad2 were quantified by densitometry using IMAGEJ analysis software. Relative p-Smad2 level was normalized compared to Smad2 level. Values are expressed as mean ± sd (** *p* < 0.03, n = 3). (**E**) Confocal study showed the nuclear localization of p-Smad2 in the fibroblasts of patient (Pt) and control (Ctrl) lines, with or without Losartan incubation (200 µM) for 14 days, and staining with anti-p-Smad2 antibody. White line, scale bar 1 μm. (**F**) We analyzed the intensity of the Alexafluor 568 signal by measuring the relative intensity of pixels representative for each regions of interest (ROI) corresponding to a single cell by LAS-X software. The graph reports means ± s.d. of p-Smad2 intensity values from 100 cells for each experiment (** *p* < 0.03, n = 3).

Emerging evidence indicates that losartan, an inhibitor of angiotensin-II type 1 receptor, is capable of reducing fibrosis in vivo and in vitro [34] by reducing the canonical and non-canonical TGF-β pathway activation status [35,36,37]. Based on these experimental data, we hypothesized that losartan could be used to decrease the TGF-β signaling hyperactivation observed in patient’s fibroblasts and consequently attenuate its fibrotic profile. In order to test our hypothesis, we monitored the TGF-β signaling status by evaluating the levels of p-ERK1/2 and p-Smad2 in our patient’s cells treated with losartan. Immunoblotting analysis revealed a significant reduction of endogenous p-ERK1/2 and p-Smad2 in treated cells, compared to untreated fibroblasts (Figure 5A–D). Finally, to support these data, we quantified the nuclear localization of p-Smad2 in treated and untreated patient’s cells by confocal analysis. The nuclear p-Smad2 signal resulted reduced in treated cells with respect to untreated cells (Figure 5E,F). Taken together, these data suggest that the losartan induces the reduction of the TGF-β signaling pathways’ activation status in the patient’s fibroblasts.

## 3. Discussion

Here, we reported our results on a set of investigations aimed at exploring the etiopathogenesis of segmental SSS in a 12-year-old female. Recognizing segmental SSS remains an elusive task also for the experienced clinician, as demonstrated by the clinical history of this patient, who received the diagnosis after years of disease progression. As documented in the literature, segmental SSS is an exclusion diagnosis based on the combination of typical findings at examination, a history of slow progression with thickening of the dermis and worsening limitation of the affected joints, radiographs excluding an underlying hyperostosis, normal serum markers, and histology documenting an absence of skin inflammation [4,38]. In this work, we excluded that segmental SSS rose from a germline or somatic deleterious variant or intragenic rearrangement of *FBN1*. In a single patient with segmental SSS, testing for germline variants in *FBN1* were all negative [14]. More recently, a single patient with segmental SSS and negative results for *FBN1* molecular testing has been shown to carry the heterozygous c.532_543dup, p.(Phe178_Glu181dup) variant in *IL-17C* [15] by whole exome sequencing. This variant was shared with her fraternal twin sister, who apparently showed a similar phenotype, and her unaffected mother. No functional study was carried out in order to associate this finding with the phenotype and to explain the presumed lack of penetrance in the mother. Further investigations are needed to better explore the role of *IL-17C* changes in the etiopathogenesis of segmental SSS.

Here, we molecularly analyzed the dermal fibroblasts of a segmental SSS patient. By employing several approaches, we found an overactivation of Smad-dependent and non-Smad-dependent TGF-β signaling pathways, increased expression of key pro-fibrotic genes, reduced MMP2 activity, and increased fibroblast migration and proliferation compared to control cells. Finally, we evaluated the efficacy of the TGF-β signaling antagonist losartan to attenuate the TGF-β activation status in patient’s fibroblasts.

The pathogenesis of SSS is unclear. Proposed mechanisms resulted in increased acid mucopolysaccharide deposition in the dermis [39], an increased accumulation of collagen [40,41], and an inflammatory process [38]. Immunofluorescence and electron microscopy studies on biopsy specimens from SSS patients revealed an abundance of myofibroblasts and an overproduction of collagen [42]. Recently, Loeys and colleagues reported 4 families with generalized SSS and carrying *FBN1* heterozygous variants in exon 37, which encodes for the fourth TB domain of fibrillin 1 involved in mediating cell–matrix interactions and the integrin signaling event [12,43]. These variants cause the disruption of interactions between Fibrillin 1 and ECM. Consequently, this results in altered TGF-β signaling, which, in turn, leads to an excessive production and deposition of microfibril glycoproteins. Mouse lines harboring analogous germline amino acid substitutions in fibrillin-1 recapitulate aggressive skin fibrosis, which is prevented by integrin-modulating therapies and reversed by antagonism of the pro-fibrotic cytokine TGF-β [44,45]. Hence, the pathogenesis of generalized SSS due to specific germline heterozygous *FBN1* variants is documented by the effect that these variants have on the TGF-β activation. Although our work excluded that segmental SSS was due to germline and somatic *FBN1* variants in this patient, a similar mechanism can be postulated also in the localized form of SSS. Accordingly, the present study may represent a starting point for understanding the ethiopathogenesis of this condition.

In our patient, qPCR study and cellular localization analysis showed higher levels of *FBN1* transcript and tissue accumulation of Fibrillin 1, respectively. Although the molecular mechanisms generating such increased expression levels of *FBN1* remain unclear in our patient, we could identify in such a preliminary finding a phenocopy of the presumed overactivation of the TGF-β pathway in generalized SSS. Since we failed to identify any *FBN1*-associated variants in our patient, other mechanisms, such as epigenetic changes or abnormalities of trans-acting transcriptional factors, could be evoked to explain such excess of Fibrillin 1 and to be investigated in future studies. Many works approached the question of how an internal duplication in *Fbn1* (carrying an in-frame duplication of exons 17–40) leads to skin in tight skin mice (Tsk), which is an animal model of generalized thickening and hardening of the dermis [46,47]. Tsk mice skin shows abnormally prominent and poorly organized microfibrillar aggregates. Saito and colleagues showed that transgenic mice expressing mutant *Tsk–Fbn1* develop permanent skin fibrosis, suggesting that the intragenic duplication of *Fbn1* likely drives dermal fibrosis [48]. It has been demonstrated that Tsk fibroblasts are able to synthesize, secrete, and incorporate both normal and Tsk Fibrillin 1 into microfibrils. However, how this contributes to excess collagen deposition is unclear [48,49]. Our finding of increased expression levels of *FBN1* transcript in dermal fibrotic tissue is in line with such consolidated evidence in the Tsk mouse model.

The involvement of TGF-β into the etiopathogenesis of SSS has been proposed [50]. It is well known that the cellular response of dermal fibrosis is led by the overactivation of TGF-β-canonical and non-canonical signalings. In particular, the activation of endogenous ERK1/2 has previously been implicated in the TGF-β mediated fibrotic response in general, and specifically in fibroblasts of patients with generalized SSS [23,44]. In addition, TGF-β activation induces phosphorylation and the nuclear translocation of Smad2, which interacts with multiple co-activators and repressors generating distinct transcriptional fibrotic responses [27]. Consequently, the nuclear accumulation of phosphorylated Smad2 (p-Smad2) serves as a positive marker of the TGF-β signaling status.

Here, the TGF-β profibrotic profile was investigated via different approaches by using dermal biopsy or cell culture primary fibroblast’s patient lines. We provided preliminary evidence of a significant overexpression of pro-fibrotic genes, including *COL1A1*, *COL3A1*, *AGT*, *LTBP,* and *ITGB1*. Conversely, values for *ITGB5* were not significant. While overexpression data could explain the observed profibrotic phenotype [25], the normal (or nearly normal) values of *ITGB5* remain an issue, which is probably related to the tissue-specific role of this gene [49]. In addition, our studies revealed a reduction of MMP2 activity in patient’s fibroblasts. MMP proteins are a family of zinc-dependent endoproteinases that are known to cleave many components of the extracellular matrix, including collagens. In particular, MMP2 can degrade type I collagen, which is the main structural protein of skin, and type IV collagen, which is a major component of basement membrane [51]. Therefore, in the setting of human fibrosis, excessive collagen deposition is not only a consequence of increased collagen synthesis but also suggests attenuated collagen degradation [52]. Thus, our results suggest that an altered MMP2 activity may contribute to alter the balance between collagen synthesis and degradation, which in turn contributes to the development of fibrosis.

The alteration of TGF-β signaling results is sufficient to initiate the fibrosis program in localized scleroderma (morphea) and systemic scleroderma [53]. In these disorders, it has been demonstrated that TGF-β induces the differentiation of fibroblasts into smooth muscle cell-like myofibroblasts, collagen secretion into the extracellular space, macrophage activation, and immune cell infiltration [54]. Taken together, our data define a tissue and cellular pattern of anomalies that confirms segmental SSS as a fibrotic localized skin disorder, and we identify in the overactivation of TGF-β and downstream alterations a key pathogenic factor of this disorder. The close similarities with generalized SSS were not supported by the identification of a somatic *FBN1* variant in our patient, but the documented overexpression of *FBN1* still supports some form of commonality between generalized and segmental SSS.

A more in-depth understanding of molecular and cellular events underlying fibrosis might allow the development of novel therapies for an effective treatment of fibrosing skin diseases [52]. At this time, no specific treatments are available to reverse or attenuate the disease manifestation and progression of SSS. A number of treatments have been tried in individual cases, including steroids, immunosuppressant drugs, psoralens (light-sensitizing medications), and light therapy. These treatments have not been helpful in slowing or stopping disease evolution [55]. A paper discussed the possibility of using mycophenolate mofetil, which is a medication that suppresses the immune system, in combination with physical therapy to treat segmental SSS [14]. In a further work, secukinumab, a humanized IL-17A antibody, was used to treat a patient with segmental SSS and carrying a variant in *IL-17C* [15]. A third paper empirically proposed the assumption of losartan in a single pediatric patient with segmental SSS [16]. In this case, a 2-year treatment was reported as effective in attenuating disease manifestations. The proposed efficacy of losartan in segmental SSS is in line with our findings and, more in general, with the fibrotic nature of this disorder. In our preliminary data, we observed a clear improvement of Smad2 and ERK1/2 proteins in localized SSS fibroblasts and demonstrated that losartan treatment reduces the nuclear localization of p-Smad2 staining. Whether this cellular phenotype might reflect or not disease amelioration after losartan treatment in a clinical setting remains to be investigated in appropriate study designs. Nevertheless, our preliminary results could represent a rationale for future studies exploring experimental therapies in segmental SSS.

## 4. Materials and Methods

### 4.1. Patient and Samples

The patient signed an informed consent to participate to this study, and she underwent blood sampling and skin biopsy for the following investigations. This study is in accordance with the 1984 Helsinki declaration and its following modifications, and the conservation and use of biological samples for scientific purposes were approved by the local ethics committee (protocol no. GTB12001).

### 4.2. Cell Cultures

The patient’s fibroblasts were derived from a skin punch biopsy of a lesional area of the right scapula. Control samples were obtained by skin biopsies from the deltoids of two unaffected individuals. Primary cell lines were cultured in Dulbecco’s Modified Eagle’s medium /Nutrient Mixture F/12 (DMEM/F12) (Thermo Fisher Scientific, USA), plus 10% fetal bovine serum (FBS, Thermo Fisher Scientific, Waltham, MA, USA) and 1% streptomycin and penicillin (Thermo Fisher Scientific, Waltham, MA, USA). Cells were grown in a 5% CO_2_ incubator at 37 °C. Primary dermal fibroblasts cultures were deposited into Genomic and Genetic Disorders Biobank (GGDB) [56] including control fibroblasts, which were established from the skin biopsies of two healthy and unrelated individuals. Skin fibroblast cell cultures were used at low passage (P1–P2) in each experiment. For all passages, between 1 and 2 × 10^6^ cells were seeded into T25 flasks and grown to 80–90% confluence.

### 4.3. DNA Extraction and Next-Generation Sequencing

Genomic DNA was extracted from peripheral blood and cultured fibroblasts by using Bio Robot EZ1 (Qiagen, Hilden, Germany), according to standard procedures. The DNA was quantified with a Nanodrop 2000 C spectrophotometer (Thermo Fisher Scientific, Waltham, MA, USA). DNA underwent next-generation sequencing (NGS) with a custom-made HaloPlex gene panel (Agilent Technologies, Boulder, CO, USA) designed to selectively capture known genes associated with syndromic and non-syndromic thoracic aneurysms and/or syndromes with Marfanoid habitus, and including: *ACTA2* (NM_001141945), *BGN* (NM_001711.5), *CBS* (NM_000071.2), *COL1A1* (NM_000088), *COL1A2* (NM_000089), *COL3A1* (NM_000090), *COL4A1* (NM_001303110), *COL5A1* (NM_000093), *COL5A2* (NM_000393), *DLG4* (NM_001365.4), *EFEMP2* (NM_016938), *ELN* (NM_000501), *EMILIN* (NM_007046.3), *FBN1* (NM_000138), *FBN2* (NM_001999), *FLNA* (NM_001110556), *FOXE3* (NM_012186), *GATA5* (NM_080473), *LOX* (NM_002317), *LTBP3* (NM_001130144.2), *MAT2A* (NM_005911.5), *MED12* (NM_005120.2), *MFAP5* (NM_003480), *MYH11* (NM_001040113), *MYLK* (NM_053025), *NOTCH1* (NM_017617), *PLOD1* (NM_001316320), *PRKG1* (NM_006258), *SKI* (NM_003036), *SLC2A10* (NM_030777), *SMAD2* (NM_005901.5), *SMAD3* (NM_005902), *SMAD4* (NM_005359), *SMS* (NM_004595.5), *TAB2* (NM_015093), *TGF-B1* (NM_000660.6), *TGF-*β*2* (NM_001135599), *TGF-*β*3* (NM_003239), *TGF-*β*R1* (NM_001306210), *TGF-*β*R2* (NM_001024847), *UPF3B* (NM_080632.2), and *ZDHHC9* (NM_016032.3). Then, targeted fragments were sequenced on a MiSeq platform (Illumina, San Diego, CA, USA) using a MiSeq Reagent kit v3 (600-cycle). Pre-processed fragments were aligned with the human reference genome hg19 (GRCh37), quality measures comprising the specificity and sensitivity of the capture, enrichment, and per-target read coverage were calculated by means of TEQC (https://doi.org/doi:10.18129/B9.bioc.TEQC) besides variants were identified by GATK [57] and annotated with ANNOVAR (http://annovar.openbioinformatics.org/en/latest/). Data analysis was performed considering the frequency, impact on the encoded protein, conservation, and expression of variants using distinct tools, dbSNP (https://www.ncbi.nlm.nih.gov/snp), 1000 Genomes (http://www.internationalgenome.org), and ExAC (http://exac.broadinstitute.org). Candidate variants were filtered according to the guidelines of the American College of Medical Genetics and Genomics (ACMGG/AMP) [58]. In order to exclude any somatic mosaic variants, DNA extracted from patient’s fibroblasts was analyzed followed by NGS analysis by applying a sensitive sequencing assay with mean coverage depth per base of approximately 3700X.

### 4.4. CytoScan XON

Chromosome microarrays analysis on patient’s DNA extracted from cultured skin fibroblasts was performed using the CytoScan™ XON array (Thermo Fisher Scientific, Waltham, MA, USA). This array contains 6.85 million empirically selected probes for whole-genome coverage including 6.5 million copy number probes and 300,000 single-nucleotide polymorphism (SNP) probes for loss of heterozygosity analysis, duo-trio assessment. The sensitivity of the platform is 95% for the detection of exon-level CNVs. The CytoScan™ XON assay was performed according to the manufacturer’s protocol, starting with 100 ng of patient DNA. This test was focused at excluding intragenic microdeletions/duplications involving genes coding for the components of the TGF-β pathway listed in the NGS-targeted panel described above.

### 4.5. Immunohistochemistry Analysis

Immunohistochemical analysis was performed on the automated platform Autostainer Link 48 (Dako, Carpinteria, CA, USA) according to the manufacturer’s instructions. Briefly, 3-µm thick formalin fixed paraffin-embedded tissue sections were deparaffinized in xylene, rehydrated in graded alcohols, washed in double-distilled water, and incubated with monoclonal anti-CD34 antibody (prediluted, clone QBEnd 10, Dako, Glostrup, Denmark) and anti-FBN1 antibody (1:100 dilution, Thermo Fischer, Waltham, Massachusetts, USA) for 20 and 30 min, respectively. Anti-FBN1 antibody was treated with DAKO solution (EnVision FLEX Target Retrieval Solution, High pH) for antigen retrieval. The primary antibodies were detected by using a commercially available kit (EnVisionTMFLEX+, Dako, Glostrup, Denmark) following the manufacturer’s protocol and diaminobenzidine as chromogen. Finally, the sections were counterstained with Mayer’s hematoxylin and mounted with Biomount (BIO-OPTICA, Milan, Italy).

### 4.6. RNA Extraction and Reverse Transcription

Total RNA was extracted from cultured skin fibroblasts with the RNeasy Mini Kit (Qiagen, Tübingen, Germany), treated with RNase free-DNase (Qiagen, Tübingen, Germany), quantified by Nanodrop (Thermo Fisher Scientific, Waltham, MA, USA), and reverse-transcribed with the QuantiTect Reverse Transcription Kit (Qiagen, Tübingen, Germany), according to the manufacturer’s protocol.

### 4.7. Quantitative Real-Time PCR

RNA extracted from patient’s and controls’ fibroblasts were processed for qPCR. Oligos for qPCR were designed using the Primer express program [59]. Primers were checked both by Basic Local Alignment Search Tool (BLAST) and BLAT (BLAST-like alignment tool) against the human genome to ensure specificity. Reactions were run in triplicate in 10 µL of final volume with 10 ng of sample cDNA, 0.3 mM of each primer, and 1x Power SYBR Green PCR Master Mix (Thermo Fisher Scientific, Waltham, MA, USA). Reactions were set up in a 384-well plate format and run in an ABI Prism7900HT (Thermo Fisher Scientific-Applied Biosystems, Carlsbad, CA, USA). Raw cycle threshold (Ct) values were obtained using SDS 2.4 (Applied Biosystems, Carlsbad, CA, USA). Calculations were carried out by the comparative Ct method as reported in [60]. Significance was determined by a two-tailed unpaired *t*-test for means. *EEF1A1* and *18S rRNA* genes were used as references for “Ct value” normalization.

### 4.8. Western Blotting and Quantification of Phosphorylated Proteins

Skin fibroblast cell cultures from patient and controls at 2 passages were seeded (2 × 10^5^ cells/mL in six-well plates) and after 24 h analyzed for Western blotting assay. Fibroblasts were lysed in 1× Dulbecco’ s Phosphate-Buffered Saline (D-PBS), 0.025% NP-40, protease inhibitors (Roche, Pasadena, CA, USA) and phospho-inhibitors (Roche, Pasadena, CA, USA). Supernatant was cleared by centrifugation. Proteins were resolved by electrophoresis on 10% SDS-gel, followed by transfer to nitrocellulose membrane. Membranes were probed with the following Abs: anti-Vimentin (1:1000, #5741S, Cell Signaling, Dellaertweg, The Netherlands), anti-β Actin (1:1000, sc-4777, Santa Cruz, USA), anti-ERK1/2 (1:1000 dilution, #4695, Cell Signaling, Dellaertweg, The Netherlands), anti-p-ERK1/2 (1:1000 dilution, #4370, Cell Signaling, Dellaertweg, The Netherlands), anti-NFkB (1:1000 dilution, #8242, Cell Signaling, Dellaertweg, The Netherlands), anti-Smad2 (1:500 dilution, #3122, Cell Signaling, Dellaertweg, The Netherlands), anti-p-Smad2 (1:500 dilution, #3108, Cell Signaling, Dellaertweg, The Netherlands), anti-MMP2, (1:1000 dilution, #PC342 Merck-Millipore, Milan, Italy), and anti-GAPDH (1:1000 Santa Cruz, CA, USA) Abs. To analyze the reactive ERK1/2 or Smad2 phosphorylation level, the band intensity of phosphorylated p-ERK1/2 or p–Smad2 and total ERK/1 or Smad2 was quantified using Image J software, and the ratio of p-ERK1/2 or p–Smad2 to total ERK1/2 or Smad2 was calculated. Fibroblast cell lines were treated or not with TGF-β1 (10 ng/mL, #T1654-1UG, Sigma, Aldrich, Milan, Italy) during 2 h in a serum-free medium before being analyzed.

### 4.9. Confocal Study

For microscopy analysis, the patient and control fibroblast cells at 2 passages were plated in 12-well plates (1 × 10^5^ cells/mL) and 24 h after, they were treated or not with TGF-β (10 ng/mL) during 2 h in serum-free medium, before being analyzed. After treatment, cells were subjected to immunofluorescence analysis, as previously reported [61]. Cells were counterstained with anti-FBN1 Ab (1:100 dilution, #PA5-27358, Thermo Fischer, USA) and anti-p-Smad2 Ab (1:200 dilution, #3108, Cell Signaling, Dellaertweg, The Netherlands) for 2 h at room temperature followed by incubation with Alexa Fluor 568 goat anti-rabbit Ab (1:500, #A11011 Thermo Fisher Scientific, Waltham, MA, USA). Cells were examined on a Leica TCS SP8 confocal microscopy (Leica, Wetzlar, Germany). All confocal images were obtained using the necessary filter sets for Alexa Fluor 568 using a X40 (1.2 numerical aperture) water immersion objective. The acquisition of data was performed with the same intensity settings and quantified measuring the relative intensity of pixels representative for each ROI corresponding of a single cell by eLAS X software (Leica, Wetzlar, Germany).

### 4.10. Zymography

First, 50 µg of non-reduced protein samples from patient’s and controls’ fibroblasts cell lines, diluted in non-reducing Laemmli buffer (62.5 mM Tris-HCl, pH 6.8, 20% glycerol, 0.5% bromophenol blue; all from Sigma Aldrich, Milan, Italy) were resolved by 10% acrylamide SDS-gels, containing type A gelatin from porcine skin (0.2%, Sigma Aldrich, Milan, Italy) as reported in [62]. Briefly, after electrophoresis, gels were incubated with a solution of 2.5% TRITON X-100 for 3 h at room temperature, and then, they were incubated in a solution of 1 mM calcium chloride and 15 mM sodium chloride, pH 7.4 (all from Sigma Aldrich, Milan, Italy) overnight at 37 °C. Subsequently, gels were fixed and then stained with Coomassie Blue (Sigma Aldrich, Milan, Italy) in order to reveal gel bands hydrolyzed by gelatinases. Following gelatin zymography, gels containing samples run in triplicate were subjected to densitometric analysis to quantify the relative MMP2 activity levels of the patient with respect to two controls.

### 4.11. PrestoBlue Assay

Cell viability was measured using PrestoBlue reagent (Thermo Fischer, Carlsbad, CA, USA) and according to the manufacturer’s protocol [63]. Briefly, skin fibroblasts cells (1 × 10^4^ cells per well) were seeded in 12-well plates (BD Falcon, Franklin Lakes, NJ) with a transparent bottom and incubated overnight. After 24 h, PrestoBlue reagent was added to determine the starting point of cell viability assay after cells were seeded. Plates were incubated for 30 min at 37 °C and 5% CO_2_. Fluorescence at 560 excitation and 590 nm emission was determined using a QuantusTM Fluorometer (Promega, Southampton, UK). The cell viability was determined after 24 h and 48 h. PrestoBlue endpoints were determined in triplicate for each replicate. T-tests were performed to compare the differences between the patient and control cell lines, and a *p*-value < 0.05 was considered statistically significant.

### 4.12. Transwell Migration Assay

For Transwell migration assay, 9 × 10^4^ fibroblast cells from patient and controls were seeded in the upper compartment of a 24 well-format Transwell with 8 µm pores (Corning, Amsterdam, The Netherlands) in 250 µL of DMEM/F-12 with 20% FBS. In the lower compartment, 1 mL of DMEM/F-12 with 20% FBS was added. Cells were incubated at 37 °C in a saturated atmosphere at 5% CO_2_ for 1, 3, and 6 h. After the incubation, cells on either face of the porous membrane were fixed by incubation with formalin 4% for 20 min at room temperature. Then, cells were stained with a 1 µg/mL solution of 4′,6-diamidin-2-fenilindolo (DAPI) for 1 min at room temperature. Once stained, cells on the upper side of the porous membranes were gently removed using a cotton swab. Then, the Transwell inserts were placed under a fluorescence microscope and images of different fields (n = 9) were collected at 20× magnification. To assess the migration rate, for each condition, stained cells were quantified by using the ImageJ program.

### 4.13. Losartan Treatment

Losartan potassium salt (Sigma Aldrich, Milan, Italy) was dissolved into dimethyl sulfoxide (DMSO) (Sigma Aldrich, Milan, Italy) and used at the concentration of 200 µM for 14 days on controls’ and patient’s fibroblast cultures as reported in [64,65]. Skin fibroblast cell cultures from the patient and control at 2 passages were seeded (2 × 10^5^ cells/mL in six-well plates) and after 24 h were incubated with losartan in a single dose in DMEM/F-12 with 20% FBS medium. After treatment, fibroblasts were processed for immunoblotting and for confocal analyses.

### 4.14. Statistical Analysis

A densitometry analysis of immunoblots was performed using ImageJ and Adobe Photoshop software. Statistical analysis of immunoblots was performed using an unpaired, two-tailed Student’s *t*-test (Excel software) (* *p* < 0.05, ** *p* < 0.03, *** *p* < 0.01). For proliferation assay, statistical analysis was performed using an ANOVA test, with *p* < 0.05 considered statistically significant.

## 5. Conclusions

In conclusion, we demonstrated, for the first time, overactivation of the profibrotic activity and TGF-β signaling in segmental SSS and suggested that the similarities between generalized and segmental SSS do not reflect a shared molecular causative event. If supported by other studies, this work could open the path to the identification of a biomarker for segmental SSS as well as novel therapeutic resources in this condition.

## Abbreviations

CNVs	copy number variations
COLL	collagens
Ctrl	control
D-MEM	Dulbecco’s Modified Eagle Medium
ECM	extracellular matrix
ERK	extracellular signal-regulated kinase
FBN1	fibrillin-1
LTBP	Latent transforming growth factor beta binding protein
MMP2	Matrix metalloproteinase 2
MRI	Magnetic Resonance Imaging
NGS	next generation sequencing
NF-κB	Nuclear factor κ-B
PBS	Phosphate-buffered saline
Pt	patient
qPCR	quantitative polymerase chain reaction
ROI	region of interest
SDS-PAGE	sulfate: polyacrylamide gel electrophoresis
SSS	stiff skin syndrome
TGF-β	Transforming growth factor β
Tsk	Tight skin mice
